# Prevalence and correlates of depression and depression-anxiety in patients receiving gastroscopy

**DOI:** 10.3389/fpsyt.2025.1585083

**Published:** 2025-08-28

**Authors:** Fan Hua, Liu Chang, Li Xin Chun

**Affiliations:** Department of Psychiatry, The Second People’s Hospital of Hunan Province (Brain Hospital of Hunan Province), Changsha, Hunan, China

**Keywords:** depression, depression-anxiety, prevalence, organic gastrointestinal diseases, upper gastrointestinal

## Abstract

**Objective:**

There are increasing numbers of patients suffering from gastrointestinal disorders who are at primary risk for depression. These patients often have no awareness of their depression and therefore choose to see their gastroenterologists. Normally gastroenterologists advise the patients to undergo gastroscopy to investigate their possible digestive disorders while overlooking their depression. This study investigated the prevalence of the comorbidities between depression, depression-anxiety and organic diseases of upper gastrointestinal tract (UGI) among patients receiving gastroscopy in a large general hospital in China.

**Methods:**

A total of 707 patients who agreed to recommendation for gastroscopy were investigated using the nine-item Patient Health Questionnaire (PHQ-9). The patients of PHQ-9 scores ≥10 were further interviewed using the Hamilton depressive scale (HAMD), Hamilton anxiety scale (HAMA) and DSM-IV to confirm the diagnosis.

**Results:**

Altogether, 412 patients were found to suffer from organic diseases of UGI based on the gastroscopy results. Of these, 51 patients and 34 were diagnosed with major depression and depression-anxiety respectively. The detection rate of depression by gastroenterologists was 3.92% while no depression-anxiety was diagnosed. Multiple logistic regression showed the course of disease, number of gastroscopies and age were significantly associated with major depression while educational level, income, age, and number of gastroscopies were significantly associated with depression-anxiety.

**Conclusion:**

The comorbidities rates between depression, depression-anxiety and organic diseases of UGI are higher than the general population in China. However, the detection rates of the comorbidities by gastroenterologists are low.

## 1 Introduction

Gastrointestinal diseases are common and may often be accompanied by mental disorders, especially depression (1). Furthermore, patients with major depression are also at increased risk for comorbidity with anxiety disorders in primary care (2, 3). A high proportion of patients who complain of gastrointestinal symptoms have been found to suffer from depression and/or anxiety (4–8).One study showed that the prevalence of depression and comorbidity of depression and anxiety in gastrointestinal outpatients to range as high as 14.39% and 4.66%, respectively (9). However, many patients with gastrointestinal symptoms have been found to suffer from functional gastrointestinal disorders (FGID) rather than true digestive organ pathology. The relationship between depression, depression-anxiety and organic gastrointestinal diseases has yet to be clarified.

An increasing number of studies have found organic diseases of UGI are associated significantly with depression and anxiety. One study found inflammation may be directly involved in the pathogenesis of depression and treating the gastrointestinal inflammation may improve the depression (10). Peptic ulcer disease is also thought to be highly co-morbid with anxiety (11, 12). The risk of depression patients who suffer from chronic gastric ulcer is three times higher than among those without depression (13). Furthermore, depression and anxiety not only impair quality of life in patients with digestive tract cancers they also decrease survival time (14, 15).

Gastroenterologists typically examine patients complaining of upper gastrointestinal symptoms with a gastroscope because electronic gastroscopy is a convenient and low-priced method of precise assessment for organic disease in the esophagus, stomach and upper duodenum (16). However, mental health issues are rarely addressed.

This cross-sectional study investigated the patients experiencing gastroscopies in the Second People’s Hospital of Hunan Province in Changsha, China. The goal is to characterize the rates of psychiatric comorbidities with confirmed organic diseases including depression, depression-anxiety as well as socio-demographic and clinical correlates of the comorbidities and to compare observed rates with those identified by gastroenterologists themselves.

## 2 Methods

### 2.1 Subjects

The target population was all patients who are receive gastroscopy at the Second People’s Hospital of Hunan Province. Simple random sampling was used to obtain the sample. The subjects had to be aged 18 years and above, to have finished the gastroscopy, completed the questionnaire and agreed to participate in the research. However, the patients who have other serious physical diseases or mental illness, including language and hearing problems or ongoing psychological treatment, were excluded. About 707 gastroscopy patients were assessed by the clinic.

### 2.2 Data collection and measurements

The research uses a cross-sectional study method carried out in the Second People’s Hospital of Hunan Province. We randomly choose one gastroscope and interviewed patients examined by that equipment. The patients completed socio-demographic data and the PHQ-9 after finishing the gastroscopy after 5 minutes recovery time.

Patients with PHQ-9≥10 were then interviewed by two psychiatrists using the Hamilton depression scale, the Hamilton anxiety scale and applying DSM-IV criteria to confirm the diagnoses. Neither the patients nor the psychiatrists knew the gastroscopy results prior to the assessments.

### 2.3 Measures

The instruments used in the study include the nine-item Patient Health Questionnaire (PHQ-9) (17), the self-report basic circumstances questionnaire (documenting sex, age, education status, income, result of the endoscopy, etc.), the Hamilton depressive scale (HAMD) (18) and the Hamilton anxiety scale (HAMA) (19). The PHQ-9 is a self-report questionnaire which has 9 items addressing psychiatric symptoms, scaled 0-3 points for each. It is used as a coarse screen to identify patients who may have had depression in the past two weeks. The scores≥10 is considered positive. The Hamilton depressive scale and the Hamilton anxiety scales are used to assess symptom severity and DSM-IV criteria were used by two psychiatrists to confirm the diagnosis of depression or depression-anxiety.

### 2.3 Data analysis

Date analysis was analyzed using the Statistical Package for the Social Sciences 16. Chi-square tests (χ 2) were used to compare the significance of differences in categorical date while *t* tests were used to compare continuous variables between individuals with and without depression and depression-anxiety disorders. Date that did not conform to a normal distribution was compared using nonparametric tests. Multiple logistic regressions were used to further analyze the association between independent demographic data and clinical characteristics (independent variables) and the diagnosis of major depression or depression-anxiety (dependent variable). Significance levels were set at 0.05. The lack of data was treated as a missing value and the observation was excluded.

## 3 Results

### 3.1 Prevalence of depressive and depression-anxiety disorders

A total of 707 cases were collected in this study. Among them, 295 patients showed no obvious abnormalities in gastroscopy. 412 patients were suffering from organic disease include erosive inflammation, peptic ulcer, polyp, tumor, and other less frequent diagnoses. **Table 1** showed that 22.5% (93/412) of patients with confirmed organic GI disease screened positive. According to DSM-IV criteria, 51(12.38%) patients were diagnosed major depression while 34(8.25%) patients were diagnosed depression-anxiety. The mean PHQ-9 score of the depression and depression-anxiety were 13.18 ± 3.98 and 14.00 ± 4.44, respectively. In the positive patients, 17.64% and 8.82% of them were in the range of moderate or severe depression and depression-anxiety according to the Hamilton scale. None of the depression and depression-anxiety patients had received any psychiatric treatment, and only 3.92% of patients meeting criteria for major depression had been advised by their gastroenterologist to see a psychiatrist.

**Table 1:** Prevalence of major depressive and depression-anxiety disorders in patients with organic diseases of UGI.

	frequency (n=412)	$\bar{\text{X}}$ ±SDa	MAHD≥17/MAHA≥14 (%)b	advise(%)
PHQ-9≥10	93(22.57)	12.10±3.36	9.68/ 3.22	—
major depression	51(12.38)	13.18±3.98	17.65	3.92
depression-anxiety	34(8.25)	14.00±4.44	32.35	0.00

a Score of PHQ-9 in patients with organic diseases of UGI

b Proportion of patients with score of Hamilton depressive scale≥17 or Hamilton anxiety scale≥14

To further clarify the prevalence of depression and depression-anxiety in different pathological types of upper gastrointestinal organic diseases, we roughly classify upper gastrointestinal organic diseases into erosive inflammation, peptic ulcers, polyps, tumors, and other uncommon lesions according to different pathological classifications. **Table 2** showed that proportions of major depression, depression-anxiety and no mental disorders in different pathological classifications. The largest proportion with major depression and depression-anxiety was found in patients with erosive inflammation.

**Table 2:** Proportions of major depression, depression-anxiety and no mental disorders in different pathological classifications.

	Erosive Inflammation	ulcer	polyp	tumor	others	total
Major depression	23(45.10)	15(29.41)	5(9.80)	3(5.82)	5(9.80)	51
depression-anxiety	17(50.00)	9(26.47)	1(2.94)	2(5.88)	5(14.71)	34
no mental disorders	136(41.59)	93(28.44)	28(8.56)	18(5.50)	52(15.90)	327
total	176	117	34	23	62	412

### 3.2 Demographic and clinical characteristics

We used t-tests or non-parametric tests to analyze whether there were differences in continuous measurement data such as age, family economic income, years of education, disease duration, and the times of gastroscopy examinations between patients with upper gastrointestinal organic diseases combined with and without major depression and depression-anxiety. The chi-square test was used to analyze the differences in categorical variables such as gender, marital status, and employment status. Patients diagnosed with major depression and depression-anxiety were more frequent in younger patients with high incomes, high levels of education and who received repeated gastroscopies (**Table 3**). However, there were no significant differences between patients with and without major depression in gender, marital status and employment status as well as depression-anxiety.

**Table 3:** Characteristics of patients with organic diseases of UGI with and without major depression and depression-anxiety.

Characteristic	no mental disorder	major depression			depression-anxiety		
	$\bar{\text{X}}$ ±SD	$\bar{\text{X}}$ ±SD	t/u	p	$\bar{\text{X}}$ ±SD	t/u	p
age income education	41.96±10.87 2200.77±1270. 8.76±3.80	35.57±10.3 2755.88±1773.63 10.47±3.2	3.932 -2.151 -3.035	<0.001 0.038 0.003	36.82±10.74 2963.24±1963.15 10.41±3.403	2.626 -2.219 -2.429	0.009 0.033 0.016
	mean rank	mean rank			mean rank		
times of gastroscopy	185.29	216.47	-1.979	0.048	251.59	-2.386	0.017

Logistic regression indicated that age, course of disease, frequency of past gastroscopy were significantly associated with the diagnosis of depression while age, level of education, incomes, frequency of gastroscopy were significant associated with depression-anxiety (**Table 4**). Age is the only protective factor.

**Table 4:** Logistic regression of characteristics of patients with major depression and depression-anxiety.

	Major depression		Depression-anxiety	
	OR	P	OR	P
age	0.924	0.002	0.941	0.034
course of disease	1.010	<0.001	1.003	0.409
times of gastroscopy	1.663	<0.001	2.003	<0.001
level of education	1.121	0.065	1.185	0.025
incomes	1.000	0.062	1.000	0.020

## 4 Discussion

### 4.1 Prevalence of major depression and depression-anxiety in general hospitals or gastroenterology dept

We examined data on 412 patients with established organic diseases of UGI (including chronic inflammation, peptic ulcer, tumor, polyp and so on). The prevalence of major depression was 12.38% and the depression-anxiety was 8.25% according to the DSM-IV criteria assessed by 2 psychiatrists. These rates were substantially higher than the prevalence of major depression (6%) in China in the general population as reported by WHO in 1999. There have been further studies on the prevalence of depression and depression-anxiety in the general population in other countries as well. Major depression ranged from 3% to 16.9% across countries (20–22). Besides, the prevalence of major depression in internal medicine clinic patients of general hospitals was reported ranged from 3.6% to 41% and depression combined with anxiety was 3.0%-5.2% (23–27). And specifically, the prevalence in patients treated by gastroenterology departments ranged from 7.5% to 19.1%, with almost a half of them combined with anxiety (4, 6, 9, 28). Furthermore, in the small samples studied gastroenterologic intensive care units, the prevalence was reported as high as 45% (29, 30). The big discrepancy in all above studies were tightly tied to the culture, subjects, diagnostic methods, research instruments and risk factors (2, 31, 32). However, there were few studies of prevalence of major depression and depression-anxiety in patients suffering from organic diseases of UGI diagnosed by gastroscopy. One small sample studied in an indirect way reported the prevalence of psychiatric disorder among UGI patients diagnosed with organic diseases by gastroscopy was 21% (33). Our study provides data that are somewhat more credible because the diagnostic accuracy of comorbidities was assessed not only by gastroenterologists with results of gastroscopy, but also by experienced psychiatrists using standardized diagnostic instruments.

In the subjects of comorbidities between major depression, depression-anxiety and organic diseases of UGI, we found that patients suffering from chronic inflammation, peptic ulcer and tumor in upper gastrointestinal had the largest proportion of such disorders. Although it is well known that the prevalence of chronic inflammation and peptic ulcer in the upper digestive tract is high in GI diseases, the relationship of chronic inflammation, peptic ulcer and psychotic has been often ignored. Some studies found that gastritis was the major diagnoses in GI patients with depression and/or anxiety disorders (26, 34). More recent research indicates that physician diagnosed gastritis was associated with major depression compared to those without gastritis (35). Feher Janos et al. demonstrated that depression may be a neuropsychiatric manifestation of a chronic inflammatory syndrome and suggested that treating gastrointestinal inflammations may improve the depression itself (10). Peptic ulcer which is common in upper gastrointestinal was also associated with depression and anxiety (12, 36). Besides, a dose-response relationship exists between the number of anxiety symptoms and increased risk of peptic ulcer disease (11). One previous study reported the prevalence of depression and anxiety in duodenal ulcer to be 13.5% and 28.8% respectively which is higher than in our study (37). This may be attributable to the diagnostic methods used and subject characteristics. In addition, in spite of a smaller percentage of tumors in digestive disorders, gastrointestinal cancers are also related to Psychiatric disease. Studies of these patients reported a prevalence of depression that ranged from 21% to 57% and anxiety that ranged from 17% to 47.2% in patients with digestive tract cancer (14, 38, 39). The high prevalence of psychiatric disease in GI cancer attaches considerable importance to the comorbidity.

### 4.2 Emphasis on these comorbidities by gastroenterologists

However, although the prevalence of comorbidities is high in patients with organic diseases of UGI, the detection rate of them in ordinary GI practice is very low. In our study only 3.92% of patients with organic GI disorders were advised to seek care for psychiatric illness. Comparing with the diagnostic data from GI outpatients, our figure was similar to 4.0% in Shenyang and 4.14% in others area of China (9, 24) and was higher than 2.8% in Beijing (26). Whereas it was lower than that in United States, Taiwan and gastrointestinal ICUs (29, 40, 41). The differences of detection rates probably relates to the high prevalence of mental disorders in these areas. Overall, the diagnostic rate of mental diseases by gastroenterologists is lower than their true prevalence. The reasons for the low recognition rate are multifaceted, mainly including those of physicians, patients, and other objective factors. Regarding physicians: The physicians specializing in gastroenterology have insufficient knowledge about depression and anxiety. They do not have a sufficient understanding of comorbidity situations. The outpatient consultation time is relatively short, which easily leads to missed diagnoses. Regarding patients: We analyzed two reasons: 1. As previously mentioned, the popularization of depression and anxiety-related knowledge is insufficient, and most patients do not understand it and choose to visit non-psychiatric departments; 2. When the physical symptoms of depression overlap with upper gastrointestinal organic diseases (42), or when mental symptoms and physical symptoms occur simultaneously, even if the severity of both is at the same level, patients are more inclined to pay attention to their physical symptoms (30). Objective factors: 1. Due to cultural differences, most people in China hold discriminatory attitudes towards mental illnesses, making patients reluctant to specifically express their mental problems to physicians. 2. Upper gastrointestinal organic diseases combined with depression and anxiety are more severe, and the complexity of the disease increases, making the diagnosis more difficult. 3. When upper gastrointestinal organic diseases are combined with depression, it requires the joint treatment of gastroenterology physicians and psychiatry physicians. However, currently, only large hospitals have mental departments, and most others exist independently as separate psychiatric hospitals. Gastroenterology physicians and psychiatry physicians have less contact, making patients with combined depression unable to receive timely relevant diagnosis and treatment. Based on the above reasons, strengthening the connection between gastroenterology and psychiatry physicians, popularizing depression-related knowledge, and enhancing gastroenterology physicians’ understanding of this comorbidity have practical significance.

### 4.3 Influences of clinical characteristics and social-demographic factor on these comorbidities

The organic diseases of UGI are various and complex. In addition, physical symptoms often overlap with psychiatric symptoms, especially fatigue and poor appetite. In our study, major depression and depression-anxiety was most prevalent in younger patients with high incomes, high education and frequent gastroscopy. There are also studies that reported that major depression is highly prevalent in younger patients and high-income countries (20, 21, 43). Yet other studies demonstrate that older age, low education, and low income level were independently associated with the depression (24, 44). There is no unified view of which social-demographic factors may lead higher to prevalences of major depression and depression-anxiety. However, many studies indicate psychosocial and environmental stressors are common in young people and are not only pathogenic of the gastrointestinal diseases, but also could induce depressive performances by affecting hippocampal BDNF level (45–47),thus young patients with organic gastrointestinal diseases may common suffer depression. On the other hand, low income and educated patient know little about symptoms and prevalence of depression while high income patient are more likely to consider medical method when they experience related symptoms (48–50). For this reason, major depression and depression-anxiety in patients suffering from organic disease in upper gastrointestinal illness with high incomes and education may be highly prevalent. However, there is little research about the association between the number of gastroscopies and major depression or depression-anxiety. As we know, patients who received gastroscopy repeatedly may suffer from more severe organic diseases of UGI or may be more worried about their conditions. Such patients show more depression and anxiety.

## 5 Limitation

There were several limitations to our study. First, the study was carried out in one general hospital in the central region of China and may not represent all of China let alone Asia. Secondly, some patients who suffered from critical diseases, such as upper gastrointestinal bleeding, could not be included in the study. These patients,mental conditions were ambiguous due to severe medical conditions. Thirdly, the study only explored depression and depression-anxiety in patient with organic diseases of UGI. Further research is needed to discuss mental conditions in patients suffering from organic diseases in lower gastrointestinal tract. Fourth, which illness among depression, depression-anxiety and organic diseases of UGI is the cause and how they affect each other are still not clear.

## 6 Conclusion

This research indicates that there was high prevalence of major depression and depression-anxiety in patients with organic diseases of UGI. However, these patients rarely received drug treatment or any other psychiatric care and gastroenterologists pay little attention to their comorbidities. Strengthening mental state examination for organic diseases of UGI and improving gastroenterologists,awareness of these comorbidities is an urgent task.

**Disclosure:** The authors disclosed no proprietary or commercial interest in any product mentioned or concept discussed in this article.

## Data Availability

The raw data supporting the conclusions of this article will be made available by the authors, without undue reservation.
